# Association between pre-reversal systemic inflammation response index and low anterior resection syndrome in rectal cancer patients: a retrospective cohort study

**DOI:** 10.3389/fonc.2026.1712384

**Published:** 2026-02-23

**Authors:** Xuena Zhang, Qingyu Meng, Jingru Wang, Simeng Jiang, Zhongtao Tian, Zihan Fan, Tong Wang, Wenbo Niu

**Affiliations:** 1 Department of Nursing, The Fourth Hospital of Hebei Medical University, Shijiazhuang, Hebei, China; 2 Department of General Surgery, The Fourth Hospital of Hebei Medical Universityi, Shijiazhuang, Hebe, China

**Keywords:** bowel dysfunction, low anterior resection syndrome, preventive stoma, rectal cancer, systemic inflammation response index

## Abstract

**Purpose:**

This study aims to explore the relationship between the systemic inflammation response index (SIRI) and the development of low anterior resection syndrome (LARS) in rectal cancer patients after ileostomy closure.

**Methods:**

This retrospective cohort study included 116 rectal cancer patients who underwent low anterior resection with diverting ileostomy and subsequent ileostomy reversal at the Fourth Hospital of Hebei Medical University between August 2022 and April 2024. SIRI was calculated from complete blood counts obtained within 1 week prior to ileostomy reversal. Postoperative bowel function was evaluated using the validated LARS questionnaire at 12 months after ileostomy closure. The association between SIRI and major LARS was examined using multivariable logistic regression models. A spline-based smooth curve fitting approach was applied to assess potential nonlinearity, and subgroup analyses were performed to explore effect modification across clinically relevant strata.

**Results:**

Among the 116 patients, 47 (40.5%) developed major LARS, while 69 (59.5%) had no or minor LARS. Compared to the no/minor LARS group, the major LARS group showed significantly higher SIRI levels (P = 0.011). Multivariate logistic regression analysis indicated that elevated SIRI was associated with a 295% increased risk of major LARS (OR: 3.95; 95% CI: 1.24, 12.61; P = 0.020). Subgroup analysis revealed that this association was more pronounced in patients with a lower anastomotic height (≤4 cm), younger age (≤60 years), shorter interval to stoma closure (Tertile 1), and those who did not receive adjuvant therapy.

**Conclusion:**

SIRI may become a biomarker for identifying patients at higher risk of developing severe LARS after rectal cancer surgery. Integrating SIRI into preoperative assessments could allow for early intervention and personalized management strategies to mitigate the severity of LARS.

## 1 Introduction

Low anterior resection syndrome (LARS) is a common postoperative complication in patients undergoing sphincter-preserving surgery for rectal cancer, particularly after closure of a protective ileostomy (1). LARS is characterized by fecal urgency, fragmentation, incontinence, and frequent bowel movements, leading to substantial impairment in quality of life (2). Although various therapeutic strategies have been proposed, including dietary modifications, pelvic floor rehabilitation, transanal irrigation, and neuromodulation, few patients receive effective treatment, and the clinical response remains unsatisfactory (3–7). Therefore, identifying patients at high risk of developing LARS remains crucial for early intervention and targeted care.

A range of factors have been reported to influence the development of LARS, including low anastomotic height, duration of stoma, and exposure to radiotherapy or chemotherapy (8). In recent years, the role of inflammation has gained attention in the pathogenesis of bowel dysfunction after rectal surgery. Inflammatory processes may contribute to mucosal damage, altered colonic motility, and dysregulation of the enteric nervous system, all of which are implicated in the development of LARS (9, 10). However, the specific relationship between systemic inflammatory status and postoperative bowel function has not been well established.

The systemic inflammation response index (SIRI) has emerged as a novel composite biomarker reflecting the balance between host inflammatory activity and immune response. It has been associated with cancer progression, postoperative complications, treatment resistance, and long-term prognosis in various malignancies, including gastrointestinal cancers (11–13). Its clinical utility lies in its simplicity, cost-effectiveness, and potential for risk stratification in multiple perioperative settings.

Existing studies investigating the association between pre-reversal SIRI and LARS are scarce. This study aims to explore their potential relationship, with the goal of identifying early predictors to improve postoperative functional outcomes.

## 2 Materials and methods

### 2.1 Study population

**Figure 1:**
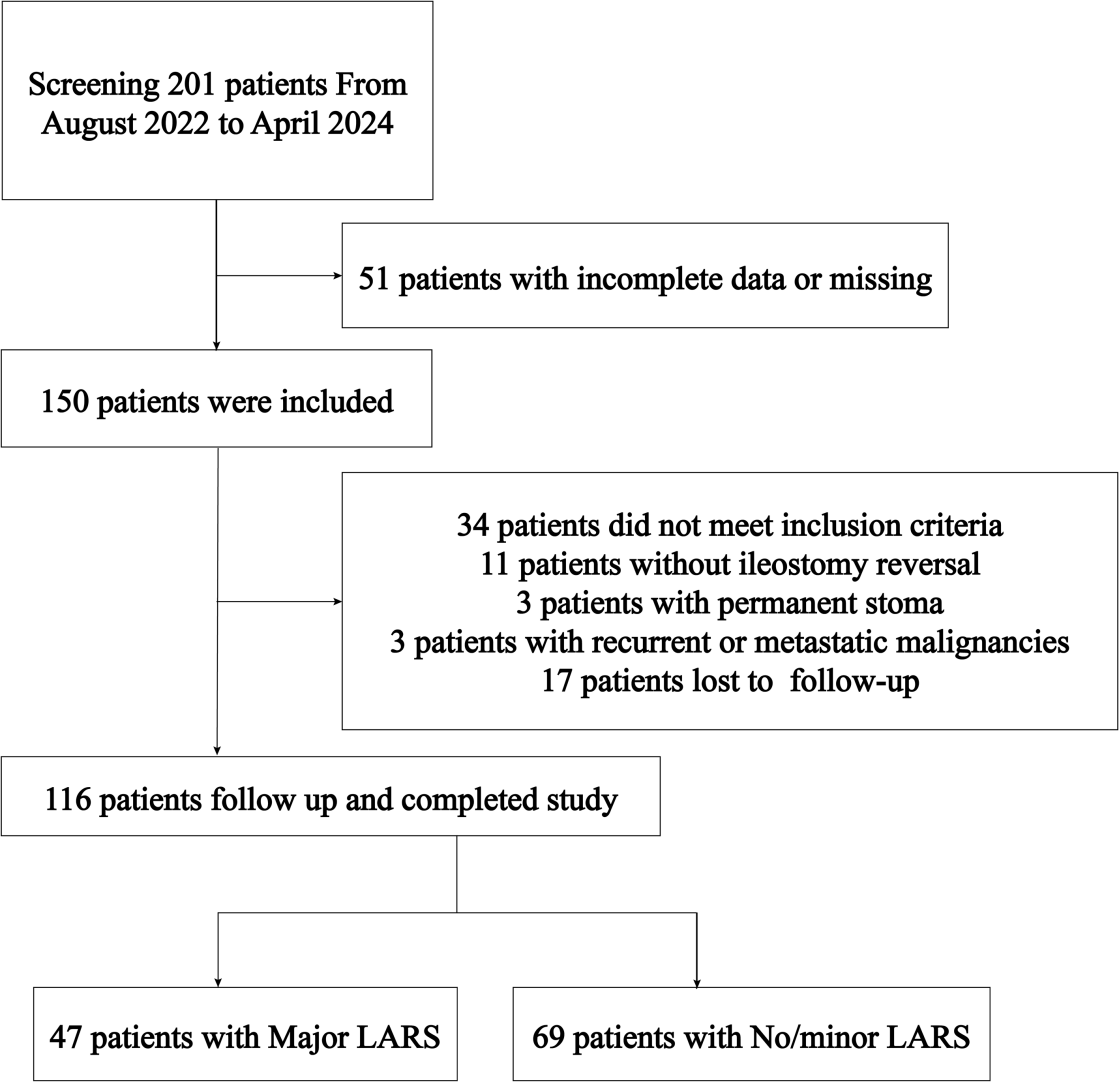
Flow chart of participants selection.

A total of 201 rectal cancer patients treated at the Fourth Hospital of Hebei Medical University between August 2022 and April 2024 were screened for participation in this study. Of these, 116 patients met the predefined inclusion and exclusion criteria. Among the eligible patients, 47 had major LARS, while 69 patients experienced no or minor LARS. Inclusion Criteria: First diagnosis of rectal cancer; Underwent anterior resection, sacral anastomosis, and prophylactic ileostomy; Received re-anastomosis following prophylactic ileostomy; Patients and their families provided consent to participate; Availability of complete clinical and follow-up data. Exclusion Criteria: Tumors located outside the rectum; Conditions affecting anal function, such as perianal abscesses or anal fissures; Severe mental disorders; Recurrent or metastatic malignant tumors; Permanent stoma; Inability to complete follow-up. Informed consent was obtained from all patients included in the study. Ethical approval was granted by the Ethics Committee of the Fourth Hospital of Hebei Medical University (Approval No. 2021111). The study flow is further illustrated in **Figure 1**.

### 2.2 Observation indicators

In this study, the LARS score was used as the primary outcome variable. Patients were categorized according to symptom severity: those with severe symptoms were assigned to the major LARS group, while individuals reporting no or only mild symptoms were placed in the no/minor LARS group. The total LARS score ranges from 0 to 42, with scores of 0–20 indicating no LARS, 21–29 indicating minor LARS, and 30–42 indicating major LARS (14). One week prior to stoma reversal, blood test parameters were collected, including neutrophil count (N) (*10^9^/L), lymphocyte count (L) (*10^9^/L), and monocyte count (M) (*10^9^/L). The SIRI was calculated using the formula: (monocytes × neutrophils)/lymphocytes (15). SIRI was categorized into two groups based on its median value. Patients with a SIRI value greater than the median were classified as High SIRI, while those with a SIRI value equal to or less than the median were classified as Low SIRI.

Based on patient demographics, surgical factors, and tumor characteristics, the following variables were observed and recorded: gender, age, postoperative TNM stage, receipt of neoadjuvant or adjuvant chemoradiotherapy, history of hypertension, cardiac disease, diabetes, and the interval between initial surgery and re-anastomosis. For patients with locally advanced rectal cancer, neoadjuvant treatment was determined through multidisciplinary team (MDT) consultation and generally included long-course chemoradiotherapy (28 fractions totaling 50.4 Gy or 30 fractions totaling 54 Gy) combined with fluorouracil (5-FU)-based chemotherapy. Surgery was performed following completion of neoadjuvant therapy. Patients who declined radiotherapy but accepted chemotherapy received neoadjuvant chemotherapy alone. Tumor height was assessed using sagittal T2-weighted magnetic resonance imaging (MRI). All imaging was interpreted by experienced radiologists and reviewed by senior imaging specialists to ensure accuracy. Prior to stoma reversal, anastomotic height was evaluated by colonoscopy.

### 2.3 Data collection

Postoperative bowel function was assessed using the LARS symptom questionnaire, which was administered during either structured telephone interviews or scheduled outpatient visits. The LARS assessment was conducted 12 months after surgery or ileostomy closure (16). Only one follow-up assessment was conducted at this time point. All participants provided written informed consent prior to enrollment. Patient eligibility was determined based on clearly defined inclusion and exclusion criteria. To ensure data reliability and reduce bias, a dedicated follow-up team was established before study initiation. Team members underwent standardized training, and consistent interview techniques were employed throughout all patient interactions to minimize interviewer influence and enhance the accuracy of self-reported outcomes. Additional clinical information, including demographic characteristics, surgical details, and imaging results, was retrieved from the institutional electronic medical records. Two independent reviewers organized and validated all clinical and follow-up data. Following consensus between reviewers, the verified data were entered into the study database.

### 2.4 Statistical methods

Normality tests were first conducted for continuous variables. As these variables did not follow a normal distribution, they were summarized as medians with interquartile ranges [M (IQR)] and compared using the Mann–Whitney U test. Categorical data were expressed as frequencies and percentages, and comparisons between binary or unordered categorical variables were performed using the chi-square test or Fisher’s exact test, as appropriate. Logistic regression analysis was employed to evaluate the association between SIRI and severe LARS. A smoothed curve fitting approach was used to assess the potential non-linear relationship between SIRI and LARS scores. Additionally, subgroup analyses were conducted to explore the association between SIRI and LARS across different population strata. Statistical significance was set at P < 0.05. All analyses were performed using Empower (R) (X&Y Solutions, Inc., Boston, MA, USA) and R software (R Project for Statistical Computing, Vienna, Austria; version 4.5.1).

## 3 Results

### 3.1 Baseline characteristics

A total of 116 patients were enrolled in the study, including 47 in the major LARS group and 69 in the no/minor LARS group. Statistically significant differences were observed between the two groups in terms of T stage (P = 0.017), tumor height (P = 0.018), anastomotic height (P = 0.002), and SIRI (P = 0.011). No significant differences were found regarding gender, age, hypertension, diabetes mellitus, heart disease, body mass index, N stage, M stage, preoperative chemotherapy, preoperative radiotherapy, or postoperative chemotherapy (**Table 1**).

**Table 1:** Characteristics of the participants.

Variables	Major LARS	No/minor LARS	*P* values
	N=47	N=69	
Gender			0.631
Male	32 (68.09%)	44 (63.77%)	
Female	15 (31.91%)	25 (36.23%)	
Age, years	63.26 (10.08) 65.00 (57.00-70.50)	61.23 (10.69) 63.00 (55.00-70.00)	0.383*
Hypertension			0.428
Yes	26 (55.32%)	33 (47.83%)	
No	21 (44.68%)	36 (52.17%)	
Diabetes mellitus			0.712
Yes	39 (82.98%)	59 (85.51%)	
No	8 (17.02%)	10 (14.49%)	
Heart diseases			0.447
Yes	43 (91.49%)	60 (86.96%)	
No	4 (8.51%)	9 (13.04%)	
Body mass index, kg/m2	24.52 (3.59) 24.77 (23.03-26.64)	24.49 (3.76) 24.49 (21.61-26.99)	0.607*
T stage			0.017
T1	3 (6.38%)	9 (13.04%)	
T2	12 (25.53%)	8 (11.59%)	
T3	4 (8.51%)	19 (27.54%)	
T4	28 (59.57%)	33 (47.83%)	
N stage			0.979
N0	32 (68.09%)	48 (69.57%)	
N1	9 (19.15%)	13 (18.84%)	
N2	6 (12.77%)	8 (11.59%)	
M stage			1.000
M0	45 (95.74%)	65 (94.20%)	
M1	2 (4.26%)	4 (5.80%)	
Preoperative chemotherapy			0.356
Yes	34 (72.34%)	55 (79.71%)	
No	13 (27.66%)	14 (20.29%)	
Preoperative radiotherapy			0.552
Yes	39 (82.98%)	60 (86.96%)	
No	8 (17.02%)	9 (13.04%)	
Postoperative chemotherapy			0.987
Yes	19 (40.43%)	28 (40.58%)	
No	28 (59.57%)	41 (59.42%)	
Stoma closure intervals, days	211.94 (84.14) 217.00 (137.00-265.50)	216.26 (111.28) 192.00 (138.00-276.00)	0.837*
Tumor height, cm	6.41 (2.63) 6.00 (5.00-8.50)	8.07 (3.58) 7.00 (6.00-10.00)	0.018*
Anastomotic height, cm	2.24 (1.52) 2.00 (1.00-3.00)	3.57 (2.35) 4.00 (1.00-5.00)	0.002*
SIRI	1.22 (0.96) 0.94 (0.69-1.25)	0.87 (0.54) 0.69 (0.52-1.12)	0.011*

The data are presented as medians (interquartile range [IQR]) and n (%). ^*^The Mann–Whitney U test was employed for the purpose of comparing two groups. LARS, low anterior resection syndrome.

### 3.2 Association between SIRI and LARS

**Figure 2:**
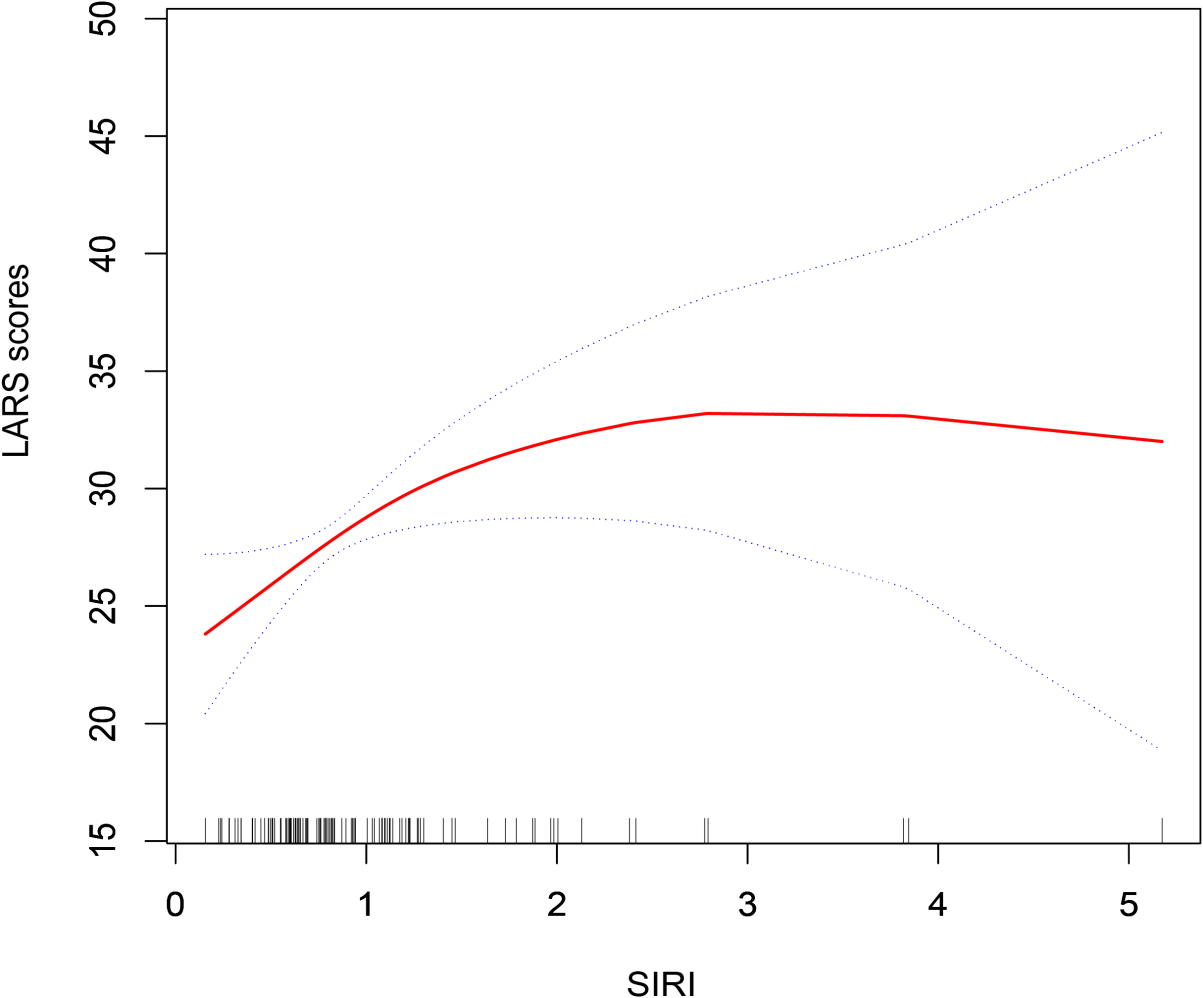
Smooth curve fitting test of the nonlinear association between SIRI and LARS. The model was fitted using Empower (R) with generalized additive models (GAM). The smooth term for SIRI was fitted with 2 degrees of freedom (edf), and the *P* value for nonlinearity was 0.0486, indicating a statistically significant nonlinear association.

Multivariate logistic regression analysis was conducted to explore the association between the SIRI and the risk of major LARS (**Table 2**). In the unadjusted model (Model I), elevated SIRI was significantly associated with an increased risk of major LARS (OR: 1.94; 95%CI: 1.08, 3.50, *P* = 0.028). This association remained statistically significant after adjusting for age, gender, and BMI in Model II (OR: 1.93; 95%CI: 1.03, 3.60, *P* = 0.039). In the fully adjusted model (Model III), which accounted for a broad range of demographic, clinical, and treatment-related covariates, the association between SIRI and major LARS remained statistically significant (OR: 3.95; 95%CI: 1.24, 12.61, *P* = 0.020). To further evaluate the nature of this association, a smooth curve fitting analysis was performed (**Figure 2**). The results revealed a nonlinear positive correlation between SIRI and LARS scores.

**Table 2:** Association between SIRI and major LARS.

Exposure	OR (95%CI)		
SIRI	Model I (unadjusted)	Model II (adjusted for age, gender, BMI)	Model III (fully adjusted)
No	Reference	Reference	Reference
Yes	1.94 (1.08, 3.50)	1.93 (1.03, 3.60)	3.95 (1.24, 12.61)
*P* values	0.028	0.039	0.020

Model I: not adjusted for any covariates; Model II: adjusted for age gender and BMI; Model III: adjusted for age, gender, BMI, T stage, N stage, M stage, preoperative radiotherapy, preoperative chemotherapy, postoperative chemotherapy, hypertension, diabetes mellitus, tumor height, anastomotic height, heart diseases and stoma closure intervals. OR, odds ratio; CI, confidence interval.

### 3.3 Subgroup analyses

Subgroup analyses were conducted to explore potential interactions between pre-reversal SIRI and relevant clinical and pathological characteristics. We evaluated the impact of SIRI on postoperative major LARS across different patient subgroups (**Table 3**). Subgroup analyses indicated a trend toward increased risk of major LARS associated with elevated SIRI among patients with lower anastomotic height (≤4 cm), younger age (≤60 years), shorter interval to stoma closure (Tertile 1), and those who did not receive radiotherapy or chemotherapy. In contrast, no statistically significant interactions were observed for gender, BMI, T stage, or N stage.

**Table 3:** Subgroup analysis of the association between SIRI and major LARS in rectal cancer patients.

Subgroup	OR (95%CI)	*P* value	*P* for interaction
Gender			0.570
Male	3.01 (0.70, 12.89)	0.137	
Female	8.19 (0.32, 213.03)	0.206	
Age (years)			0.171
≤60	11.49 (1.78, 74.11)	0.010	
>60	2.92 (0.81, 10.52)	0.102	
Hypertension			0.293
Yes	10.83 (1.00, 116.83)	0.050	
No	2.45 (0.49, 12.13)	0.274	
BMI (kg/m2)			0.390
≤24.9	5.60 (0.88, 35.54)	0.067	
>24.9	2.22 (0.56, 8.77)	0.257	
T stage			0.900
T1-2	2.43 (0.27, 21.52)	0.425	
T3-4	2.84 (0.91, 8.86)	0.072	
N stage			0.588
N0	2.65 (0.76, 9.21)	0.126	
N1-2	11.87 (0.04, 3549.89)	0.395	
Preoperative chemotherapy			0.652
Yes	3.66 (0.48, 28.01)	0.212	
No	6.67 (1.34, 33.24)	0.021	
Preoperative radiotherapy			0.457
Yes	2.06 (0.29, 14.71)	0.471	
No	6.94 (1.43, 33.62)	0.017	
Postoperative chemotherapy			0.108
Yes	3.65 (0.66, 20.04)	0.136	
No	51.07 (2.16, 1210.12)	0.015	
Stoma closure intervals (days)			0.346
Tertile 1	17.52 (1.11, 277.61)	0.042	
Tertile 2	3.16 (0.43, 23.27)	0.258	
Tertile 3	2.19 (0.50, 9.56)	0.297	
Tumor height (cm)			0.869
≤5	6.25 (1.09, 35.94)	0.040	
>5	7.62 (1.32, 44.08)	0.023	
Anastomotic height (cm)			0.017
≤4	14.27 (2.32, 87.94)	0.004	
>4	0.59 (0.05, 7.18)	0.679	

Age, gender, BMI, T stage, N stage, M stage, preoperative radiotherapy, preoperative chemotherapy, postoperative chemotherapy, hypertension, diabetes mellitus, radiotherapy, heart diseases, tumor height, anastomotic height and stoma closure intervals were adjusted. In the subgroup analyses, the model is not adjusted for the stratification variable itself.

### 3.4 Relationship between SIRI and clinicopathological characteristics

To clarify whether SIRI reflects broader systemic or tumor-related conditions, we analyzed the relationship between SIRI and clinicopathological characteristics. The analysis revealed that the High SIRI group had significantly higher proportions of males (P = 0.019), diabetes mellitus (P = 0.002), preoperative chemotherapy (P = 0.048), and preoperative radiotherapy (P = 0.018). Additionally, the incidence of severe LARS was higher in the High SIRI group (P = 0.005). No significant differences were observed between the two groups for other variables, including age, hypertension, heart disease, BMI, T stage, N stage, M stage, and postoperative chemotherapy (**Table 4**).

**Table 4:** Relationship between SIRI and clinicopathological characteristics.

Variables	High SIRI	Low SIRI	*P* values
	N=58	N=58	
Gender			0.019
Male	44 (75.86%)	32 (55.17%)	
Female	14 (24.14%)	26 (44.83%)	
Age, years	63.00 (57.00-70.00)	64.00 (54.25-70.75)	0.744*
Hypertension			0.577
Yes	27 (46.55%)	30 (51.72%)	
No	31 (53.45%)	28 (48.28%)	
Diabetes mellitus			0.002
Yes	15 (25.86%)	3 (5.17%)	
No	43 (74.14%)	55 (94.83%)	
Heart diseases			0.377
Yes	8 (13.79%)	5 (8.62%)	
No	50 (86.21%)	53 (91.38%)	
Body mass index, kg/m2	24.97 (23.10-27.38)	24.22 (21.51-25.95)	0.061*
T stage			0.350
T1	5 (8.62%)	7 (12.07%)	
T2	8 (13.79%)	12 (20.69%)	
T3	15 (25.86%)	8 (13.79%)	
T4	30 (51.72%)	31 (53.45%)	
N stage			0.716
N0	38 (65.52%)	42 (72.41%)	
N1	12 (20.69%)	10 (17.24%)	
N2	8 (13.79%)	6 (10.34%)	
M stage			0.679*
M0	54 (93.10%)	56 (96.55%)	
M1	4 (6.90%)	2 (3.45%)	
Preoperative chemotherapy			0.048
Yes	18 (31.03%)	9 (15.52%)	
No	40 (68.97%)	49 (84.48%)	
Preoperative radiotherapy			0.018
Yes	13 (22.41%)	4 (6.90%)	
No	45 (77.59%)	54 (93.10%)	
Postoperative chemotherapy			0.186
Yes	38 (65.52%)	31 (53.45%)	
No	20 (34.48%)	27 (46.55%)	
Stoma closure intervals, days	215.00 (146.25-300.25)	178.00 (125.25-259.00)	0.073*
Tumor location, cm	8.00 (5.00-10.00)	6.00 (5.00-8.00)	0.028*
Anastomotic height, cm	3.00 (1.00-5.00)	2.50 (1.00-4.00)	0.224*
LARS			0.005
Major LARS	31 (53.45%)	16 (27.59%)	
No/minor LARS	27 (46.55%)	42 (72.41%)	

The data are presented as medians (interquartile range [IQR]) and n (%). ^*^The Mann–Whitney U test was employed for the purpose of comparing two groups. LARS, low anterior resection syndrome.

## 4 Discussion

In this study, we found that a higher pre-reversal SIRI may be associated with an increased risk of major LARS in patients undergoing protective stoma closure after rectal cancer surgery. This possible association remained significant after adjusting for multiple demographic, surgical, and treatment-related factors. Moreover, smooth curve fitting revealed a nonlinear positive correlation between SIRI and LARS scores. Subgroup analyses suggested that the association was particularly pronounced in patients with a lower anastomotic height, younger age, shorter stoma closure intervals, and those who did not receive adjuvant therapy. LARS is a common complication that significantly impacts patients’ quality of life, leading to long-term functional impairment and psychological distress. This syndrome not only disrupts daily activities but also affects social functioning. Despite the availability of various therapeutic strategies, such as pelvic floor rehabilitation and pharmacological interventions, treatment outcomes remain suboptimal, making early identification of high-risk patients crucial for improving management (17–19).

Multiple risk factors have been associated with the development of LARS following sphincter-preserving surgery. Anatomical factors such as low anastomotic height, a narrow pelvic cavity, and nerve plexus injury are all linked to LARS development (20, 21). A low anastomotic height leads to greater disruption of the anorectal nerve plexus, resulting in more severe local inflammation, while also compromising the integrity of the enteric nervous system and rectal compliance, thereby increasing the risk of LARS (22–24). Similarly, a narrow pelvic cavity can increase tissue tension during surgery, leading to further inflammatory damage, while pelvic nerve injury often results in both local and systemic inflammatory responses that disturb anorectal function (25). Treatment-related factors, particularly neoadjuvant radiotherapy and chemotherapy, exacerbate the risk of LARS by inducing inflammatory responses (26). Radiotherapy triggers local inflammation and fibrosis in the pelvic region, damaging mucosal layers and impairing rectal motility, while chemotherapy suppresses immune function, potentially exacerbating tissue damage and inflammatory burden (27, 28). Additionally, the occurrence of anastomotic leaks after surgery also significantly contributes to LARS, as they lead to localized infections and inflammation, hindering healing and contributing to persistent bowel dysfunction (29). The use of preventive diverting stomas reduces anastomotic leakage but may also introduce new inflammatory burdens at the stoma site, affecting overall postoperative recovery (30). These factors converge on a common pathway of persistent low-grade inflammation, which significantly contributes to the development of LARS. SIRI has emerged as a promising biomarker for assessing the overall inflammatory burden, reflecting the balance between systemic inflammation and immune responses. Initially studied in cancer prognosis, SIRI has shown predictive value in various clinical settings, including cancer progression, treatment resistance, and surgical complications (31). Its clinical appeal lies in its objectivity, accessibility, and ability to provide a comprehensive measure of the inflammatory state, making it a valuable tool in understanding the underlying inflammatory processes of LARS.

Several mechanisms may explain the observed relationship between elevated SIRI and major LARS. SIRI integrates information from neutrophils, monocytes, and lymphocytes, providing a comprehensive reflection of both systemic inflammation and immune dysregulation. Increased neutrophil and monocyte counts indicate a persistent subclinical inflammatory state, which may promote pelvic tissue remodeling, nerve injury, and gastrointestinal motility dysfunction, all of which are critical contributors to LARS development (32–34). Elevated neutrophil and monocyte activity can lead to the release of pro-inflammatory cytokines that disrupt the normal functioning of the anorectal region, cause neuroinflammation, and promote fibrosis, leading to impaired neuromuscular coordination (35). Our analysis revealed significant associations between SIRI and factors such as age, diabetes, preoperative chemotherapy, and preoperative radiotherapy. These findings suggest that preoperative treatments and other factors may lead to an increased inflammatory response, which could exacerbate LARS, as indicated by elevated SIRI levels. This implies that SIRI may not only reflect systemic inflammation but also tumor-related factors that influence the inflammatory response. Future studies could also explore inflammation as a mediating factor in tumor treatment and LARS.

Chronic inflammation also impacts rectal compliance, primarily through the induction of fibrosis and tissue remodeling (8). Persistent inflammation can lead to the loss of normal rectal distensibility, further contributing to the functional impairment seen in LARS (22). Moreover, inflammation is increasingly recognized as a key factor in gut microbiota dysbiosis, which may exacerbate gastrointestinal dysfunction (36, 37). Inflammatory changes in the gut can disrupt the gut microbiota, leading to an imbalance that affects gut motility, visceral hypersensitivity, and the integrity of the intestinal barrier. This disruption of gut homeostasis, in turn, contributes to the structural and functional alterations in the rectum, promoting the development of LARS (38, 39).

The results of this study suggest that the SIRI, a readily available and cost-effective blood-based biomarker, has the potential to become a marker for identifying patients at high risk of developing major LARS before stoma reversal. By incorporating SIRI into preoperative risk assessments, clinicians can more accurately stratify patients, allowing for early intervention and better-informed preoperative counseling. This could guide the implementation of perioperative strategies, such as early rehabilitation and nutritional support, to help mitigate the severity of LARS in high-risk individuals. Furthermore, SIRI could enable more individualized postoperative follow-up, with enhanced monitoring and targeted interventions tailored to the patient’s inflammatory profile. Future studies should explore whether interventions aimed at reducing systemic inflammation, such as the use of anti-inflammatory therapies or lifestyle modifications, could decrease the incidence or severity of LARS when applied before stoma closure. Randomized controlled trials are needed to confirm whether these interventions can modify inflammatory pathways and improve long-term outcomes for patients at risk of developing LARS.

This study has several limitations that should be acknowledged. First, it was a retrospective, single-center analysis, which may limit the generalizability of the findings. Second, although the LARS score is a widely accepted tool, it is based on subjective patient-reported outcomes, which could introduce recall or response bias. Third, the sample size, particularly in subgroup analyses, was relatively small, potentially affecting statistical power. Fourth, this study did not specifically address the potential influence of preoperative factors such as infection, diabetes, and nutritional status on SIRI levels. These factors may confound the relationship between SIRI and the outcomes, as infection and diabetes are known to affect systemic inflammation, and nutritional status may also influence inflammatory markers. Future studies should consider these factors to better understand their impact on SIRI and its clinical significance. Finally, while we adjusted for a broad range of confounders, the possibility of residual confounding from unmeasured variables cannot be excluded. Prospective, multicenter studies with larger cohorts are warranted to validate our findings.

## 5 Conclusion

This study suggests that a higher pre-reversal SIRI is associated with an increased risk of major LARS in rectal cancer patients. Elevated SIRI reflects a systemic inflammatory state that may contribute to postoperative bowel dysfunction, offering potential for early identification of high-risk patients. While the findings support the clinical utility of SIRI as a biomarker, the limitations of this study, including its retrospective design, small sample size, and potential biases in subjective symptom reporting, must be acknowledged. Future research should focus on prospective, multicenter studies to validate these results and explore therapeutic strategies aimed at reducing systemic inflammation to improve postoperative outcomes in patients at risk of LARS.

## Data Availability

The raw data supporting the conclusions of this article will be made available by the authors, without undue reservation.
